# Factors Associated With Successful MRI Scanning in Unsedated Young Children

**DOI:** 10.3389/fped.2018.00146

**Published:** 2018-05-22

**Authors:** Camilia Thieba, Ashleigh Frayne, Matthew Walton, Alyssa Mah, Alina Benischek, Deborah Dewey, Catherine Lebel

**Affiliations:** ^1^Department of Radiology, University of Calgary, Calgary, AB, Canada; ^2^Child & Adolescent Imaging Research Program, University of Calgary, Calgary, AB, Canada; ^3^Department of Pediatrics, University of Calgary, Calgary, AB, Canada; ^4^Department of Community Health Sciences, University of Calgary, Calgary, AB, Canada; ^5^Owerko Centre, Alberta Children's Hospital Research Institute, University of Calgary, Calgary, AB, Canada; ^6^Hotchkiss Brain Institute, University of Calgary, Calgary, AB, Canada

**Keywords:** magnetic resonance imaging, mock scanner, sedation, anesthesia, children, neuroimaging

## Abstract

**Introduction:** Young children are often unable to remain still for magnetic resonance imaging (MRI), leading to unusable images. Various preparation methods may increase success, though it is unclear which factors best predict success. Here, in a retrospective sample, we describe factors associated with successful scanning in unsedated young children. We hypothesized that the mock scanner training and fewer behavior problems would result in higher success rates.

**Methods:** We recruited 134 children aged 2.0–5.0 years for an MRI study. We compared success between children whose parents opted for mock scanner training (*n* = 20) or not (*n* = 114), and evaluated demographic and cognitive factors that predicted success.

**Results:** Ninety-seven children (72%) completed at least one MRI sequence successfully on their first try; 64 children (48%) provided high-quality data for all 3 structural imaging sequences. Cognitive scores were higher in successful than unsuccessful children. Children who received mock scanner training were no more likely to be successful than children without, though they had slightly higher scores on T1 image quality.

**Conclusions:** Our data shows that scanning with minimial preparation is possible in young children, and suggests limited advantages of mock scanner preparation for healthy young children.Cognitive ability may predict success.

## Introduction

Magnetic resonance imaging (MRI) is a non-invasive technique useful for numerous research and clinical applications, though it is very sensitive to motion. Collecting high quality datasets is particularly difficult in young children (~2–4 years), as they do not readily fall asleep or follow instructions to stay still. Sedation or general anesthesia are often used in clinical settings (1, 2), but are not appropriate for research (1, 3–5). Scanning children during natural sleep can be successful (3, 6–8), but requires flexible scanning schedules (i.e., evening/night time), and can require long waiting periods for children to fall asleep. Preparation techniques for scanning awake children include training in a mock scanner and play therapy (6, 9–14); audio/visual systems can also increase compliance (5, 9, 15, 16). Success rates vary from 33 to 97% with various preparation methods (6, 12, 13, 16), though it is difficult to determine which factors most drive success.

In our ongoing study of brain development in young children, we use a rocketship themed training protocol, with optional mock scanner training. This protocol requires little preparation time and can be implemented in centers lacking flexible scanning hours. Here, we describe the protocol and evaluate factors associated with children's success. We hypothesized that mock scanner training, and fewer behavior problems would be associated with higher success rates.

## Materials and methods

### Participants

This was a retrospective analysis of data collected for a study examining brain and language development in early childhood (17). One hundred thirty-four children aged 1.96–4.95 years (3.4 ± 0.6 years) were recruited from the Calgary area. All were English speakers born full term, and free from genetic disorders associated with significant intellectual or motor impairments, neurological or neurodevelopmental disorders, and contraindications to MRI. Written informed consent was obtained from a parent/guardian. The Conjoint Health Research Ethics Board at the University of Calgary approved this study, REB13-0020.

At the time of the MRI scan, years of maternal post-secondary education was collected, as well as NEPSY-II Phonological Processing and Speeded Naming language assessments (18) on all children aged 3 years or older. Some children were part of another (non-imaging) study (19) and were assessed ~1 year prior to scanning on the Cognitive, Language, and Motor Composite scales of the Bayley Scales of Infant and Toddler Development-III (Bayley-III) (20) (*n* = 104) and the Attention, Internalizing, and Externalizing Behavior Problems scales of the Achenbach System of Empirically Based Assessment (21) (ASEBA; *n* = 96).

### Pre-scanning training sessions

During a screening call to determine eligibility, families were provided with links to audio, video, and website resources about MRI scanning, including our e-book *Pluto and the MRI Rocket Ship Adventure*, which incorporates our scanning procedures into a story (Supplementary Figure 1; https://www.lulu.com/shop/search.ep?contributorId=1347527); parents were encouraged to practice with their child. Families were also offered an optional training session on our mock scanner, which is equipped with a rocket ship façade identical to that on the real MRI scanner (Supplementary Figure 1). Only 15% (*n* = 20) of families opted for mock scanner training. The most common reason for declining was because parents thought their child did not need it. Mock scanner training occurred within the week prior to the MRI scan. Sessions lasted 30–40 min, and consisted of briefly describing the MRI procedure, introducing the child to the scanner, putting a parent or stuffed animal into the bore, and having the child practice lying still within the bore while watching a movie and listening to typical MRI scan sounds. At the end of the training, children were given an astronaut training certificate.

Families were reminded 1–2 days prior to the real MRI scan to review preparation materials. On the day of the scan, children were met by a research assistant, who played short games to make the child comfortable, completed the 10-min language assessment, read the rocketship story, and overviewed expectations for the MRI. The child was told about the “big camera” that makes loud noises, instructed to lie still while “pictures” were being taken, given stickers, and promised a gift at the end of the procedure. A teddy bear was given to each child to take with them inside the scanner, as described in our book. The family was taken to the scanner and given a brief opportunity to familiarize themselves with the environment (~5 min). The child was shown the equipment and asked to lie down on the scanner bed, where he/she was fitted with headphones and positioned inside the head coil, then inside the bore. During scanning, a parent remained beside the child, and children watched movies. Children were reminded to “hold still like a statue.” Total time spent preparing for imaging was ~15–20 min. At the completion of the scan, children were given an astronaut certificate for their “space flight,” and a toy.

### MR imaging

Imaging was performed during daytime/early evening hours (< 7 pm) at the Alberta Children's Hospital on the research-dedicated 3T GE MR750w MRI scanner (General Electric, Waukesha, WI) with a 32-channel head coil. The protocol consisted of: diffusion tensor imaging (DTI; 4:03 min), anatomical T1-weighted imaging (4:12), then T2^*^-weighted imaging (4:12). Arterial spin labeling, spectroscopy, and resting state functional MRI were acquired on participants when time permitted; these are not discussed here. Foam padding was used to minimize head motion.

#### Scan quality assessment

In-house Matlab software was used to detect DTI volumes with excessive motion, which were removed prior to data analysis. Scans with >22 volumes (66%) retained were considered successful and suitable for tractography and voxel-based analysis. T1- and T2^*^-weighted image quality was rated on a five-point scale, where 1 represented unusable data and 5 represented no motion artifacts (Supplementary Figure 2). Scans with scores of 3–5 were considered successful and suitable for further analysis. Children with high quality scans for all three sequences were considered “successful,” those with data from one or two sequences were considered “partially successful.”

### Statistical methods

Statistical analysis was performed in SPSS version 24.0. One way ANOVAs were used to test demographic, cognitive, and behavioral differences among successful, partially successful, and unsuccessful groups. Demographics and quality measures (DTI volumes retained, T1 score, T2^*^ score) were also compared between children who received mock scanner training and those who did not, using non-parametric tests for two independent samples. The association between mock scanner training and scan success was tested using a chi-squared test.

## Results

### Image quality

Results here are for the first attempt at scanning (i.e., no participants returned for a second visit). Median ratings for T1- and T2^*^-weighted images were 3; the mean number of usable DTI volumes was 31 (of 35). Sixty-four children (48%) provided high quality scans for all 3 sequences. An additional 14 children (10%) provided high quality data for 2 sequences and 19 (14%) provided high quality data for only 1 sequence; these 33 (27%) children formed the partially successful group. Thirty-seven children (28%) were unsuccessful.

### Scanning success

Group differences were observed for NEPSY-II Phonological Processing and Speeded Naming, and Bayley-III Cognitive and Language Composite scores. For Phonological Processing, and Cognitive and Language composities, the successful group scored higher than the unsuccessful group; for Phonological Processing and Speeded Naming, the successful group scored higher than the partially successful. The partially successful and unsuccessful groups did not differ on any metrics. No significant group differences were found for maternal education, or child's age, sex, or behavior (Table 1).

**Table 1:** Demographic characteristics of children participating in the study.

	**Successful (*n* = 64)**	**Partially successful (*n* = 33)**	**Unsuccessful (*n* = 37)**	***p*-value (ANOVA)**
Maternal education (post-secondary)	5.7 ± 2.5	6.2 ± 3.5	5.5 ± 2.3	0.55
NEPSY-II Phonological processing	10.1 ± 3.1^a^^,^^b^	8.4 ± 2.8	8.7 ± 3.5	**0.011**
NEPSY-II Speeded naming	10.8 ± 3.2^b^	8.9 ± 3.3	9.6 ± 3.7	**0.050**
Bayley-III cognitive composite	120 ± 16^a^^*^	113 ± 17	108 ± 14	**0.027**
Bayley-III language composite	115 ± 13^a^^*^	114 ± 14	107 ± 16	**0.039**
Bayley-III motor composite	109 ± 14	109 ± 13	106 ± 16	0.65
ASEBA attention problems	53 ± 5	52 ± 4	54 ± 6	0.68
ASEBA internalizing problems	43 ± 8	47 ± 13	45 ± 9	0.25
ASEBA externalizing problems	47 ± 9	47 ± 9	48 ± 11	0.87

*Groups are separated based on scan success, with success defined as at least one sequence with high-quality data. Two-sample t-tests were used to test for group differences*.

a *Significantly different (p < 0.05) from the unsuccessful group*.

b *Significanly different (p < 0.05) from the partially successful group*.

* *Indicates differences that survived multiple comparison correction*.

*The bold values indicate significant differences on the ANOVA*.

ANOVA results did not survive multiple comparison correction. For *post-hoc* testing, only differences between the successful and unsuccessful groups survived Tukey corrections for multiple comparisons.

### Training differences

Mock scanner training was not significantly associated with scan success. However, children receiving training had higher T1 ratings than children without mock scanner training (*p* = 0.012, Table 2). Children with mock scanner training also had lower cognitive and language scores (*p* = 0.008–0.035). No differences survived multiple comparison correction.

**Table 2:** Differences between groups receiving mock scanner training or not.

**Demographics**	**Mock scanner training (*n* = 20)**	**No mock scanner training (*n* = 114)**	***p*-value**
Maternal education (post-secondary)	6.1 ± 3.6	5.6 ± 2.6	0.57
Age	3.3 ± 0.7	3.4 ± 0.6	0.34
Sex	13m/7f	63m/51f	0.42
NEPSY-II phonological processing	9.6 ± 3.2	7.6 ± 2.4	**0.02**
NEPSY-II speeded naming	10.1 ± 3.4	9.7 ± 3.9	0.69
Bayley-III cognitive composite	101 ± 7	114 ± 15	**0.008**
Bayley-III language composite	103 ± 11	113 ± 14	**0.035**
Bayley-III motor composite	103 ± 10	108 ± 15	0.25
ASEBA attention problems	53 ± 5	55 ± 6	0.27
ASEBA internalizing problems	44 ± 10	47 ± 7	0.34
ASEBA externalizing problems	48 ± 7	47 ± 10	0.75
**OUTCOMES**			
Participants with successful T1 scans	10 (50%)	63 (55%)	0.06
Participants with successful T2* scans	9 (45%)	64 (56%)	0.35
Participants with successful DTI scans	10 (50%)	83 (73%)	0.62
At least 1 high-quality dataset	11 (55%)	86 (75%)	0.1
3 high-quality datasets	8 (40%)	56 (49%)	0.48
T1 rating	4	3	**0.012**
T2* rating	3	3.5	0.64
DTI volumes useable (#)	31 ± 2	31 ± 2	0.85

Two-sample t-tests were used to test for group differences; non-parametric tests were used for image quality ratings. *p < 0.05.

*The bold values indicate significant differences on the ANOVA*.

### Case-control comparison analysis

Because of the apparent sample bias in the mock scanner training group (lower cognitive and language scores), a case-control analysis was conducted (*n* = 34). Children in the training group were matched to children in the non-mock scanner training group on Phonological Processing scores, age and sex, and identical analyses were conducted. These matched training groups did not differ significantly on cognition, behavior, or scan outcomes (Supplementary Table 1).

## Discussion

Successful pediatric neuroimaging sessions are important for research on brain development and for clinical assessments. Here, in a sample of young children, 48% provided high quality data for all three structural imaging sequences, and 72% providing some high quality data, on the first attempt. Most children did not undergo mock scanner training, and used only at-home materials and had a short (15–20 min) preparation session immediately prior to their MRI scan. This methodology could be easily applied in an environment where scanning children during natural sleep and/or lengthy preparation sessions are not possible.

While the idea of using storytelling and imagination to engage children in scanning sessions is not unique (14), customizing training tools to engage children with a specific location and research group is a fairly new concept. Our training tools featured key elements of our scanning site and introduced children to the research staff involved in the training sessions. This technique helped to establish rapport with children and families prior to the onsite visit, possibly shortening the time required to build children's trust.

Few studies report success rates specifically in preschool children. One study had 54–71% success in 4 year-olds (12), and another reported 33% success for clinical scans in 2–4 year olds (16). Success rates across wider age ranges are 66% (6) to 97% (8, 13), though these studies required advance training visits, multiple attempts at scanning, and/or long preparation times (6–8, 13). Therefore, while more preparation may be beneficial, our results indicate that scanning of young children is also possible with less preparation.

Previous findings in adults showed mock scanner training reduced subjective distress (22). Here, in young children, mock scanner training was not significantly associated with success, even in a case-controlled analysis. However, the mock scanner training group had significantly higher ratings on T1-weighted images, suggesting a potential advantage for data quality.

Successful children had higher cognitive and language scores than unsuccessful children by 0.5–0.8 standard deviations, which is generally considered clinically significant (23), and thus may be plausibly related to children's ability to remain still for the MRI scan. The lack of significant group differences on behavior assessment measures suggests that success is not as closely related to attention or hyperactivity.

A limitation of this study is that it was not a randomized trial, and there was a sample bias among children who received mock scanner training. In the case-controlled analysis, only 17 children who received training could be matched on cognitive/language scores to children who did not receive training, resulting in a small sample. Future research using randomized designs and larger samples is necessary to ascertain the true effects of training protocols and other factors on scan success in children.

In conclusion, our results suggest that cognitive scores may help predict young children's success in MRI scanning, and show a potential advantage of mock scanner training for scan quality. Ultimately, this study shows the feasibility of conducting MRI exams in awake preschool-aged children, and demonstrates a need for further research regarding training protocols and variables that predict success.

## Ethics statement

This study was carried out in accordance with the recommendations of the University of Calgary Conjoint Health Research Ethics Board (CHREB). The protocol was approved by CHREB. All subjects gave written informed consent in accordance with the Declaration of Helsinki.

## Author contributions

CT, AF, and CL: performed data analysis and interpretation; CT, AM, MW, and AB: performed image analysis and quality assessment; AF, DD, and CL: wrote the manuscript. All authors read and approved the final manuscript.

## Data availability

The raw data analyzed for this manuscript will be made available by the authors upon reasonable request.

### Conflict of interest statement

The authors declare that the research was conducted in the absence of any commercial or financial relationships that could be construed as a potential conflict of interest. The reviewer EH and handling Editor declared their shared affiliation.

## Supplementary material

Supplementary Figure 1 Space adventure-themed training materials Space adventure-themed training materials include a children's book about an MRI rocket ship adventure **(A)**, astronaut training certificates **(B)**, and a rocket ship façade for our MRI scanner **(C)**.

Supplementary Figure 2 Image quality assessment Sample T1-weighted images (top row) and T2^*^-weighted images (bottom row) are shown for each quality rating. One represents poor image quality and 5 represents excellent. Images rated 3–5 were considered suitable subsequent analysis.

Supplementary Table 1 Case-Control analysis.
