# Supplementary figures and images for: Factors Associated With Successful MRI Scanning in Unsedated Young Children
Source: Front Pediatr. 2018 May 22;6:146. doi: 10.3389/fped.2018.00146 (PMC5972312; doi:10.3389/fped.2018.00146)

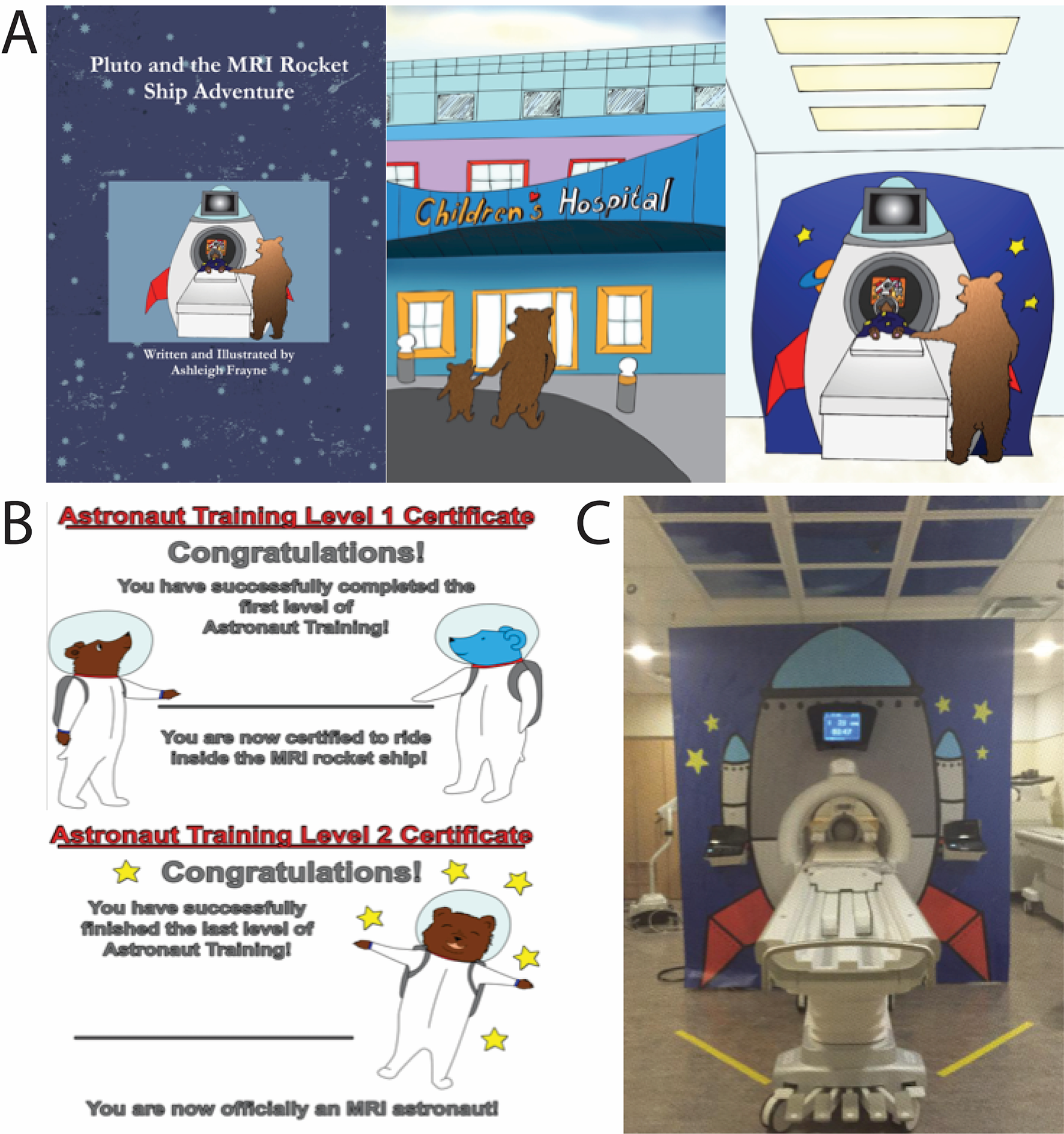

Supplement: Supplementary Figure 1 — Space adventure-themed training materials Space adventure-themed training materials include a children's book about an MRI rocket ship adventure (A), astronaut training certificates (B), and a rocket ship façade for our MRI scanner (C). [file Image_1.TIF]

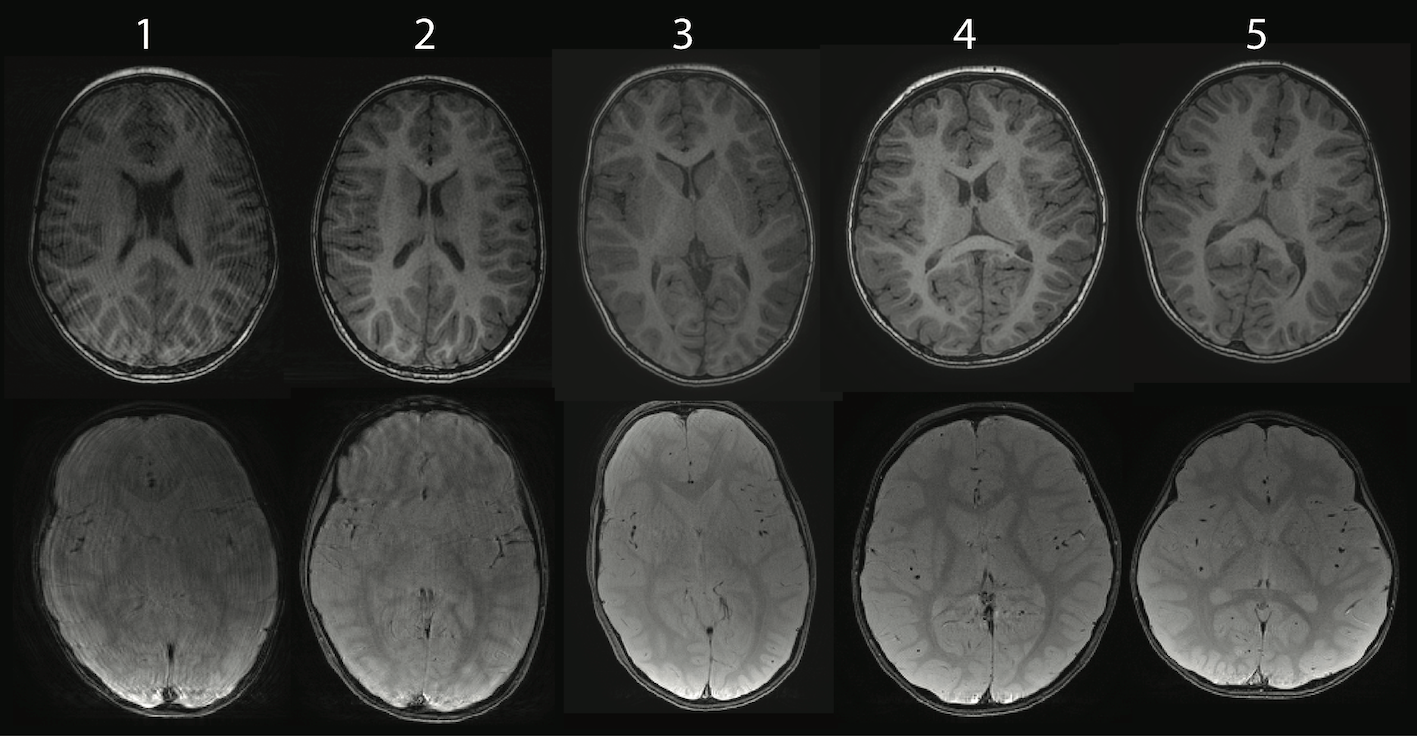

Supplement: Supplementary Figure 2 — Image quality assessment Sample T1-weighted images (top row) and T2*-weighted images (bottom row) are shown for each quality rating. One represents poor image quality and 5 represents excellent. Images rated 3–5 were considered suitable subsequent analysis. [file Image_2.TIF]
