# Supplementary material for: Factors Associated With Successful MRI Scanning in Unsedated Young Children
Source: Front Pediatr. 2018 May 22;6:146. doi: 10.3389/fped.2018.00146 (PMC5972312; doi:10.3389/fped.2018.00146)
Supplement: Supplementary Table 1 — Case-Control analysis. [file Table_1.DOCX]

**Supplementary Table 1: Case-Control analysis**

| **Demographics** | **Mock scanner training**  **(n=17)** | **No mock scanner training (n=17)** | **p-value** |
| --- | --- | --- | --- |
| **Maternal Education (y post-secondary)** | 5.9 +/- 3.7 | 4.7 +/- 1.6 | 0.28 |
| **Age** | 3.4 +/- 0.7 | 3.4 +/- 0.6 | 0.96 |
| **Sex** | 13m/7f | 13m/7f | 1 |
| **Phonological Processing** | 7.7 +/- 2.5 | 7.7 +/- 2.4 | 0.95 |
| **Speeded Naming** | 9.7 +/- 3.9 | 8.4 +/- 3.1 | 0.34 |
| **Bayley-III Cognitive Composite** | 103 +/- 7 | 106 +/- 9 | 0.42 |
| **Language Composite** | 104 +/- 12 | 107 +/- 15 | 0.58 |
| **Motor Composite** | 104 +/- 9 | 101 +/- 13 | 0.62 |
| **ASEBA Attention Problems** | 53 +/- 3 | 54 +/- 6 | 0.59 |
| **Internalizing** | 48 +/- 8 | 44 +/- 9 | 0.45 |
| **Externalizing** | 48 +/- 5 | 49 +/- 10 | 0.93 |
|  |  |  |  |
| **Outcomes** |  |  |  |
| **T1 rating** | 4 | 3 | 0.46 |
| **T2* rating** | 3 | 5 | 0.13 |
| **DTI volumes useable (#)** | 31 +/- 5 | 33 +/- 6 | 0.73 |
| **At least 1 high-quality dataset** | 7n/10y | 6n/11y | 0.79 |
| **3 high-quality datasets** | 10n/7y | 8n/9y | 0.56 |
